# Operative Surgery

**Published:** 1851-01

**Authors:** 


### ARTICLE VIII.

*Lectures on Operative Surgery in the College of Physicians and Surgeons.* By Prof. MOTT.

On Friday, Dec 13th, Professor Mott, after some concluding remarks upon the subject of Thoracic Aneurism, proceeded to speak of Ligature of the External Iliac Artery. This operation, he remarked, had been first performed by Abernethy, in 1796, but unsuccessfully. He tried it in 1806, with a successful result. He was followed by Messrs. Tomlinson and Freer, in England; while in this country it was first ligatured by Dr. Dorsey, and next by Dr. Wright Post, of this city. Afterwards by Dr. Smith, of New Haven, Dr. A. H. Stevens, Dr. Jamieson, and Dr. D. L. Rodgers, and others Dr. Mott described the different modes of operating, and then his own manner of performing it, which he exhibited at the same time upon the subject. He makes a curvilinear incision, commencing just above the external abdominal ring, and extending outwards, and parallel with Poupart's ligament, towards and a little above the anterior superior spinous process of the ilium.

The skin and superficial fascia are divided, and the tendon of the external oblique clearly exposed. This tendon is then cautiously divided to the extent of the external incision, and it is then separated from the internal oblique, and the flap turned up. The edge of the internal oblique and transversalis muscles is then carefully detached from Poupart's ligament, and turned upwards. A portion of the funnel-like, or tubular process of the fascia transversalis, which invests the cord, is then pinched up and raised by the forceps, and then divided transversely with the point of the knife. The finger is then passed into it, along the cord to the external abdominal ring. The pulsating artery is then felt behind and below the ring. The cord thus serves as a guide to the artery, while, by the above method, we are sure of getting *below* the peritonæum, so as to raise it from and above the artery. In this mode of proceeding, there is less danger of tearing, or otherwise injuring the peritonæum, than in any other plan of performing the operation. Having raised up the bag of the peritonæum, the edges of the wound are to be separated by spatulas, and by the fingers of assistants, so as to enable the operator to get as good a view as possible of the artery, which is then to be carefully separated from the vein which is below and on the inside of the artery; and only to an extent sufficient to allow of the passage of the aneurism needle. In this, as indeed in all cases, the vessel should be disturbed and isolated from its sheath as little as possible. The needle should be passed *from*, not *towards* the vein. The artery is tied generally about an inch above Poupart's ligament, and care should be taken before tying it to ascertain that no nervous filaments are included in the ligature. The edges of the wound are brought together by a suture, and slight adhesive straps, but no bandage of any kind should be applied, nor anything which may constrict the limb, or tend to interfere with its circulation. Loose cotton, or some equally good non-conductor of heat, should be placed all around the limb, from the toes to the groin, so as to cherish the heat and vitality of the part.

Dr. Mott, in speaking of the Statistics of the Operation, stated that he had ligatured this artery seven times; four times with success. Of the three remaining cases one died from secondary hemorrhage; one from peritonitis, caused by excess in the use of spirituous liquors, and the last from gangrene of the inferior extremity. This was a case of traumatic aneurism, in which the aneurismal sac communicated with the femoral vein.—*N. Y. Register of Medicine and Pharmacy.*

